# Microcystins Across the Lake–River Continuum: Transport, Transformation, Exposure, and Ecological Risk

**DOI:** 10.3390/toxins18100433

**Published:** 2026-10-08

**Authors:** Yucong Li, Xiang Wan, Fangqun Gan

**Affiliations:** 1 School of Environmental and Ecology, Jiangsu Open University, Nanjing 210017, China; 2 Jiangsu Engineering and Technology Centre for Ecological and Environmental Protection in Urban and Rural Water Environment Management and Low Carbon Development, Nanjing 210017, China; 3 College of Resources and Environmental Sciences, Nanjing Agricultural University, Nanjing 211800, China; 4 School of Chemical Engineering and Materials, Changzhou Institute of Technology, Changzhou 213032, China

**Keywords:** microcystins, cyanobacterial harmful algal blooms, lake–river continuum, hydrological connectivity, biogeochemical transformation, multi-matrix exposure, ecological risk assessment

## Abstract

Microcystin (MC) risk is often assessed within bloom-affected lakes and reservoirs, although hydrological connectivity can extend exposure into downstream river and estuarine networks. We synthesize evidence using a lake–river continuum framework that treats connected reaches as active processing environments. The framework separates the source pool, export flux, and downstream exposure, and distinguishes advective redistribution, reversible retention, irreversible transformation, and source or remobilization processes. Hydrology sets reaction opportunity, while phase partitioning, photochemical and microbial transformation, sediment exchange, and biological uptake determine how MC mass and bioavailability change during transport. Field evidence shows that downstream occurrence cannot be inferred reliably from visible source blooms: reaches may attenuate, retain, redistribute, or locally renew MC exposure depending on hydraulic state and process coupling. These dynamics shift exposure among water, particles, sediments, cells, and biota. Consequently, source-lake indicators or water concentrations alone can mischaracterize ecological exposure, and drinking-water guideline values are not ecological protection thresholds. Key priorities are flux-resolved monitoring, direct field estimates of process rates and transformation products, chronic ecotoxicology matched to realistic exposure routes, and hierarchical transport–biogeochemical models. This continuum perspective links source, process, pathway, receptor, and effect within a single source-to-risk framework.

## 1. Introduction

Cyanobacterial harmful algal blooms (cyanoHABs) are a persistent water-quality problem in eutrophic freshwaters. Global satellite and synthesis studies report increasing bloom frequency or intensity in many large lakes, with nutrient enrichment remaining a major driver and warming further favouring bloom development in many systems [1,2,3,4]. Microcystins (MCs) are among the most widely monitored cyanotoxins because of their broad occurrence and established ecological and human-health hazards [5,6]. Lake Taihu illustrates the management challenge: recurrent blooms remain a long-term concern, and the 2007 crisis disrupted drinking-water supply for more than two million residents of Wuxi [7,8].

The hazard does not necessarily end at the lake boundary. Hydrological connectivity can transport MCs and toxin-producing cyanobacterial biomass into downstream river networks and, in some systems, estuarine or coastal waters. Widespread MC contamination has been documented in the river network downstream of Lake Taihu [9]; freshwater-derived MCs have also been detected in estuarine shellfish [10], and land-to-sea transport was linked to microcystin intoxication in southern sea otters [11]. Broader syntheses confirm that freshwater-to-marine transfer occurs across multiple regions [12]. These observations establish connectivity as an exposure pathway, but they do not imply that toxin concentration or ecological risk necessarily declines or increases monotonically with distance from the source.

Connected river reaches are therefore more than passive conduits. Relative to source lakes, they differ in flow velocity, hydraulic residence time, light climate, suspended-particle load, sediment exchange, and biofilm habitat. A recent Lake Taihu lake–river comparison showed clear functional divergence between lake and river compartments, with flow acting as an important ecological filter [13]. These differences can alter not only how much MC is transported but also whether it remains dissolved, is retained by particles or sediments, undergoes transformation, or reaches different biological receptors.

We consequently treat the lake–river continuum as a dynamic biogeochemical reactor in which hydrology governs transport opportunity, reversible retention redistributes MC among environmental pools, photochemical and microbial reactions transform parent toxin, and local production or remobilization can renew the mobile signal [13,14,15]. This review focuses on MCs because continuum-scale evidence is substantially richer for this toxin class than for most other cyanotoxins; MC-LR is discussed where congener-specific toxicological or regulatory evidence requires it. The synthesis follows a compact source-to-risk sequence: source pool and export, reactor processing, receptor-specific exposure and effects, and continuum-aware risk assessment and management. The central question is not simply how far MC travels but when transport, retention, transformation, and remobilization change downstream exposure.

## 2. From the Source Pool to Downstream Export

### 2.1. Source Pool and Export-Relevant Properties

Microcystins are cyclic heptapeptides with numerous structural congeners [16,17,18]. For continuum-scale transport, three properties matter most: sufficient persistence for hydrological export, separation between intracellular and extracellular pools, and redistribution among dissolved, particle-associated, sediment-associated, and biotic compartments [19,20,21]. The relative importance of these pools changes over a bloom cycle as intact cells are transported, damaged, or lysed and as extracellular toxin interacts with particles and sediments. Congener composition and water chemistry can further modify behaviour; MC-LR hydrophobicity is pH-dependent [21], and biological degradation can differ among congeners [22]. These properties mean that neither bloom biomass nor a single dissolved-water measurement fully describes the MC inventory available for export.

Source strength is controlled by both bloom development and toxin production. Nutrient enrichment is a major driver of cyanoHAB occurrence [3,23], but MC production varies with producer composition, cellular toxin quotas, toxin release, and degradation [24,25]. Chlorophyll a or phycocyanin can therefore identify bloom conditions without directly quantifying exportable toxin. Climate forcing can alter the timing and magnitude of these source conditions: recent evidence links heatwaves and extreme precipitation to greater bloom persistence and eutrophication pressure [26], while warming may redistribute future MC hotspots [27]. These studies support greater variability in source conditions but not, by themselves, larger downstream toxin fluxes. Demonstrating export requires toxin concentration and phase to be paired with discharge at the lake–river boundary.

### 2.2. Export Flux and Hydrological Connectivity

Continuum assessment should separate the source pool, export flux, and downstream exposure. The source pool describes the amount and composition of MC available at the source; export flux is the mass crossing a hydrological boundary per unit time; and downstream exposure reflects subsequent mixing, transformation, storage, tributary input, and local production [20]. For an adequately mixed cross-section, instantaneous export can be estimated as F = QC, where Q is discharge and C is the corresponding MC concentration in consistent units; cumulative export requires integration over time. A high concentration under weak outflow can therefore generate less export than a lower concentration under stronger outflow. Reservoir-release observations illustrate why source-water concentrations need not represent outlet or downstream conditions [28], whereas warm, low-discharge conditions can favour local cyanobacterial accumulation [29].

Field evidence can be organized into three hydrological configurations. First, managed lake-to-river transfer regulates the entry of lake water and suspended biomass into receiving channels. In the Taihu–Liangxi system, a field-calibrated phytoplankton model attributed more of the annual chlorophyll-a signal to physical transport than to local proliferation [30]; this is biomass-transport evidence, not a direct MC mass balance. A separate Taihu watershed survey reported intracellular MC up to 14.5 µg/L in the lake, while river concentrations were generally below 1.0 µg/L [13]. These observations establish spatial heterogeneity but not a universal downstream removal rate or a quantified response to individual pumping and sluice operations.

The 2011 Milford Lake release provides a direct source-to-river example. Total MC in outflow never exceeded 20 µg/L and was generally less than 10% of concentrations in lake surface accumulations, yet MC was detected throughout a 173-mile (approximately 278 km) Kansas River reach within one week of the initial releases [28]. The event demonstrates downstream exposure without resolving the separate contributions of dilution, retention, and transformation.

Second, connected river–lake chains redistribute cyanobacterial populations across environmental gradients. Sequencing along the Thames River–Lake St. Clair–Detroit River–Lake Erie continuum supports biological connectivity and environmental selection [31], but shared cyanobacterial sequence variants do not quantify toxin flux. Complementary chemical and molecular evidence comes from the 2012 Klamath River survey: source tracking linked downstream *Microcystis* to Iron Gate Reservoir, and total MC concentrations of 165 µg/L in Copco Reservoir and 3.6 µg/L in the lower estuary documented toxin occurrence across a reservoir–river–estuary system [32]. Those site concentrations are not a matched water-parcel mass balance and cannot be converted into a removal percentage.

Third, downstream channels can become bloom-supporting habitats. Recurrent *Microcystis aeruginosa* blooms in the regulated Moselle during 2017–2020 coincided with warm, reduced-discharge summers. Median intracellular MC concentrations were 0.17 µg/L in August and 0.11 µg/L in September, with MC-LR, MC-RR, and MC-YR predominating [29]. These filter-extract measurements do not represent dissolved MC or a total export balance, and the imported and locally produced fractions remain unresolved.

Hydrological effects on community composition must also be distinguished from effects on species richness and toxin concentration. For matched summer periods in 2021 and 2022, a later Moselle study reported mean discharges of 328 and 39 m^3^/s and modelled flow times of 9.9 and 31.8 days, respectively; *Microcystis* bloomed in the drier, warmer 2022 [33]. That study did not report analytical MC concentrations. Because temperature and nutrient conditions also differed, it does not isolate a flow effect. The cases summarized here document assemblage shifts, biomass changes, or toxin occurrence, rather than standardized tests of species richness against discharge [13,29,31,33]. They therefore do not establish a universal monotonic flow–richness or flow–MC relationship.

**Figure 1:**
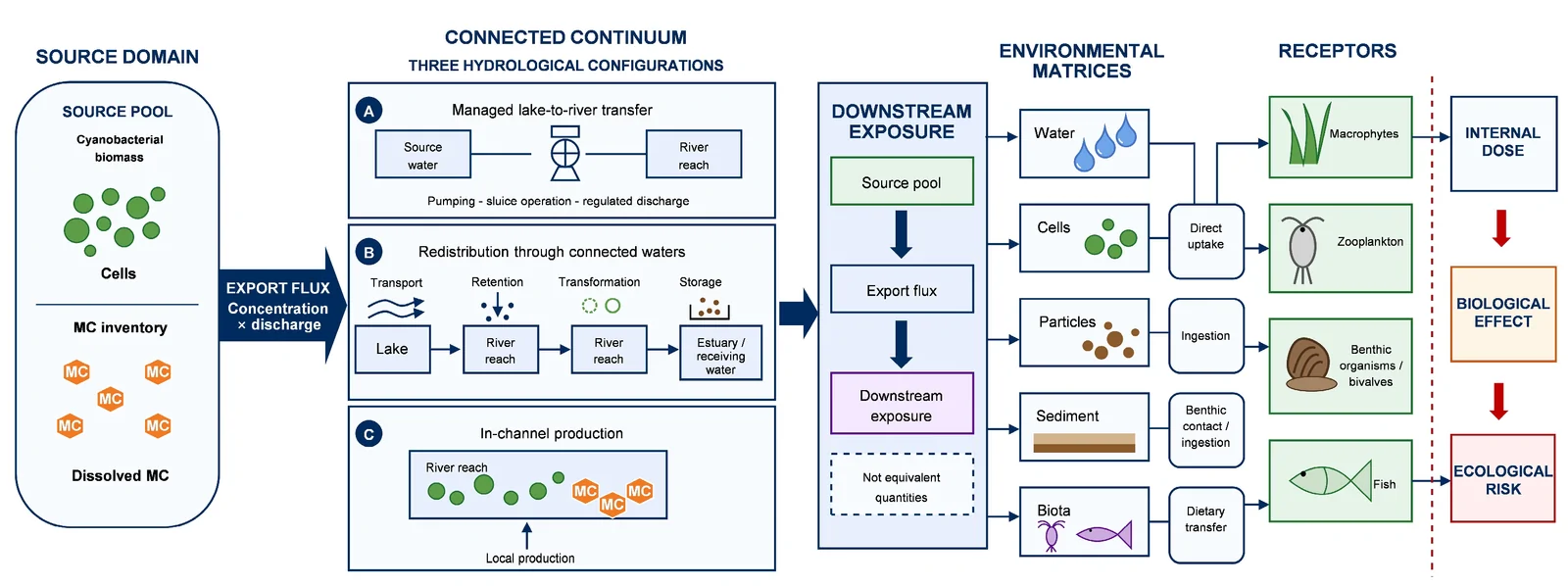
Hydrological configurations linking source pools to downstream microcystin exposure across the lake–river continuum. The framework distinguishes the source pool, export flux, and downstream exposure as related but non-equivalent quantities. The source pool comprises cyanobacterial biomass and the microcystin (MC) inventory available for export, whereas export flux depends jointly on MC concentration and water discharge. Three hydrological configurations are illustrated: (**A**) managed lake-to-river transfer, in which pumping, sluice operation, or regulated discharge controls transfer from the source waterbody to downstream river reaches; (**B**) redistribution through connected waters, in which transport, retention, transformation, and storage progressively modify the source signal during passage through connected river and receiving-water compartments; and (**C**) in-channel production, in which local cyanobacterial growth generates additional MC within downstream river reaches. Downstream exposure is distributed among water, cyanobacterial cells, particles, sediments, and biota, corresponding to different uptake pathways, including direct uptake, ingestion, benthic contact or ingestion, and dietary transfer. These matrix- and pathway-specific exposures can generate different internal doses among macrophytes, zooplankton, benthic organisms and bivalves, and fish, ultimately contributing to biological effects and ecological risk. Arrows indicate conceptual pathways rather than quantitative fluxes. The dashed box highlights the distinction among source pool, export flux, and downstream exposure, while the red dashed vertical line separates environmental/receptor exposure from organism-level internal dose, biological effect, and ecological risk.

At the downstream end of some continua, MC transport extends across the freshwater–marine boundary. Land-to-sea transfer has been demonstrated through sea-otter intoxication, marine-mussel accumulation, and estuarine shellfish surveys [10,11,12,34]. These cases establish connectivity as an exposure pathway rather than an uninterrupted transport rule. Salinity transitions, particle dynamics, residence time, degradation, and biological retention can modify the signal before it reaches coastal receptors. Together, the studies support process-level conclusions about connectivity and phase-specific exposure, whereas concentration ranges, dominant taxa, and the relative importance of local production remain system- and event-specific. Table 1 separates direct MC measurements from community or hydrological evidence and states the limits of each inference. Figure 1 summarizes the hydrological configurations.

### 2.3. Temporal Decoupling and Sediment Storage

Downstream exposure can become spatially and temporally decoupled from visible bloom conditions at the source. Cell lysis changes intracellular and dissolved fractions, advective travel delays arrival, and temporary storage in low-velocity zones, particles, or sediments changes the timing of detection. Lake Taihu observations show that lake bloom intensity is an incomplete proxy for riverine toxin conditions [13]. The Kansas release study likewise illustrates a downstream exposure window that cannot be inferred from source-lake surface accumulations alone [28]. Hydrology can decouple concentration from flux through the joint effects of discharge and concentration, whereas low-flow periods can favour residence and local cyanobacterial accumulation [29,33]. Consequently, a downstream decline in concentration may reflect dilution, tributary mixing, sorption, settling, or true degradation, whereas a flat or increasing profile may reflect local production, storage, or remobilization. Longitudinal gradients are therefore mechanistically ambiguous unless paired with discharge, tracers, or hydrodynamic information.

Sediments add a storage and memory component to the continuum. Conceptual mass-balance work places sediments alongside water-column, macrophyte, and consumer pools [20], and field studies confirm intracellular and extracellular MC variants in sediments and pore waters [35] as well as long-term sediment-associated MC records in Lake Taihu [36]. These observations establish storage, not necessarily an active secondary source. Resuspension or pore-water exchange should be treated as remobilization pathways until sediment-to-water flux is measured. Sediments can also retain viable *Microcystis*: benthic recruitment by physical resuspension has been demonstrated in lakes [37], and benthic populations can seed recurrent blooms in engineered aquatic systems [38]. The field importance of this pathway in natural river networks remains poorly quantified.

## 3. The Lake–River Continuum as a Dynamic Biogeochemical Reactor

The reactor concept is useful only if it separates processes that merely redistribute MC from those that reduce parent-toxin mass or renew the mobile inventory. Four functions are therefore distinguished: advective redistribution and dilution; reversible retention in particles, sediments, biofilms, or biota; irreversible photochemical or microbial transformation; and source/remobilization terms. Local cyanobacterial production adds new MC mass, whereas cell lysis, desorption, and resuspension primarily return existing MC to mobile or dissolved phases. Only irreversible transformation, permanent burial, or export across a defined control-volume boundary represents unambiguous loss from that parent-MC inventory. Concentration attenuation, parent-MC mass attenuation, and ecological-risk attenuation are thus distinct outcomes [19,20].

### 3.1. Hydrology and Reaction Opportunity

Hydrology determines how much time and interfacial contact are available for reaction. Dilution can lower concentration without destroying mass; high discharge may therefore reduce local concentration while increasing downstream transport and spatial reach, whereas low discharge increases residence time and contact with particles, biofilms, and sediments. Backwaters, impoundments, side channels, and low-velocity margins further create strong residence-time heterogeneity within a single reach [13,19,20]. A useful heuristic is the relation between transit time and transformation time: transport dominates when transit is much faster, while reactive processing becomes increasingly important as the two timescales converge.

Longer residence time, however, does not guarantee lower exposure. It can increase photochemical, microbial, and sediment interactions, but it can also promote local cyanobacterial growth or allow stored toxin to re-enter mobile phases [20,29]. The contrasting Moselle summers illustrate how longer modelled flow time can coincide with bloom development rather than simple removal [33]. Hydrology therefore acts primarily as a control on reaction opportunity rather than as a removal mechanism itself. The same physical reach can function differently under flood, normal-flow, drought, or managed-release conditions.

### 3.2. Reversible Retention and Phase Redistribution

Sorption and phase partitioning are retention processes, not degradation. MC-LR hydrophobicity varies with pH [21], and SPM, biofilms, and sediments can shift toxin between dissolved and particle-associated pools. In turbulent-water experiments, SPM–biofilm aggregates reduced aqueous MC-LR through adsorption, biosorption, and biodegradation while remaining biologically active toward *Daphnia* [39]. Retention can therefore reduce dissolved exposure yet increase particle ingestion or delivery to depositional zones. Sedimentation may extend residence at microbially active interfaces, but retained toxin can later return to the water column through desorption or resuspension. Biological uptake likewise transfers MC to a new compartment rather than eliminating it.

Matrix complexity makes the direction of these effects conditional. Microplastics, for example, can sorb microcystins, but adsorption depends strongly on polymer type and weathering state [40]. They are therefore better regarded as conditional partitioning surfaces than universal vectors. More generally, particle load alters several processes at once: it changes sorption capacity and microbial habitat while also modifying light penetration. Such interactions are central to the reactor concept because a change in phase can alter which subsequent transformation pathway has physical access to the toxin.

### 3.3. Irreversible Transformation

Photochemical and microbial reactions can destroy the parent MC molecule, but their field contribution depends on physical access to the reactive environment. Natural-light studies demonstrate substantial MC phototransformation, and a recent surface-water microcosm reported a summer MC-LR half-life of approximately one day under combined direct and indirect photochemical processes [41,42]. Such rates cannot be transferred directly to whole river columns because optical depth, mixing, sensitizers, and time in the photic zone differ among reaches [41,43]. Dissolved organic matter can both promote indirect photochemistry and suppress it by attenuating light or scavenging reactive species, making its net effect dependent on concentration, composition, and pH [43,44].

Microbial degradation provides a second irreversible pathway. The mlrABCD pathway remains the best-characterized mechanism [14], but community-scale degradation cannot be inferred from mlr genes alone. Lake Erie and Lake Taihu metatranscriptomes revealed candidate non-mlr responses that still require experimental confirmation [45], while metabolomic and metatranscriptomic work with Lake Erie beach sand demonstrated MC-LR degradation accompanied by diverse transformation reactions [46]. Biofilms can couple retention and transformation: river-origin phototrophic biofilms removed dissolved MC more rapidly after prior cyanobacterial exposure [47], and constructed-wetland mesocosms demonstrate removal by indigenous microbial consortia [48]. These studies support localized transformation hotspots, but disappearance of a targeted parent congener is not, by itself, evidence of complete detoxification.

Transformation products require separate chemical and biological evaluation. Natural-water phototransformation of MC-LR generates products through reactions including oxidation and bond cleavage [42]. In that study, neither the initial nor the transformed mixtures induced mutagenicity in the tested *Salmonella* strains; this negative Ames result does not establish the absence of phosphatase inhibition, hepatotoxicity, or chronic ecological effects. Parent-congener analysis should therefore be paired, where feasible, with product identification and endpoint-specific bioassays rather than interpreting analytical disappearance as unqualified risk reduction [42,49].

Biotic conjugation and binding introduce additional analytical pools. Cysteine and glutathione conjugates have been quantified in exposed carp tissues [50], and thiol conjugation can be reversible under experimental conditions [51]; neither observation establishes an environmental release rate. Covalently protein-bound MC in tissues is also distinct from freely extractable toxin. Oxidative methods can recover an integrated signal from free and bound forms, but lose congener resolution and require matrix-specific recovery controls [52]. These conjugated or protein-bound forms must not be confused with cell-associated MC retained in intact cyanobacteria. Targeted parent-MC measurements alone may miss relevant forms, whereas a larger chemically measured bound pool does not itself imply an equivalent bioavailable dose. The evidence for the main processes and their field uncertainties is summarized in Table 2.

### 3.4. Process Coupling and Dynamic Reactor States

Net attenuation emerges from process interaction rather than from independent removal rates. Turbulence can increase contact with SPM–biofilm aggregates but also maintain particles in suspension [39]; high particle loads increase sorption surfaces while reducing light penetration; and sorption can remove MC from the photic dissolved pool while increasing residence at microbially active interfaces. A clear, shallow reach may favour photochemistry but provide little retention time, whereas a turbid low-flow reach may suppress photolysis while enhancing particle and biofilm interactions. These compensating and reinforcing effects explain why a single laboratory-derived attenuation coefficient is unlikely to describe a heterogeneous river network.

**Figure 2:**
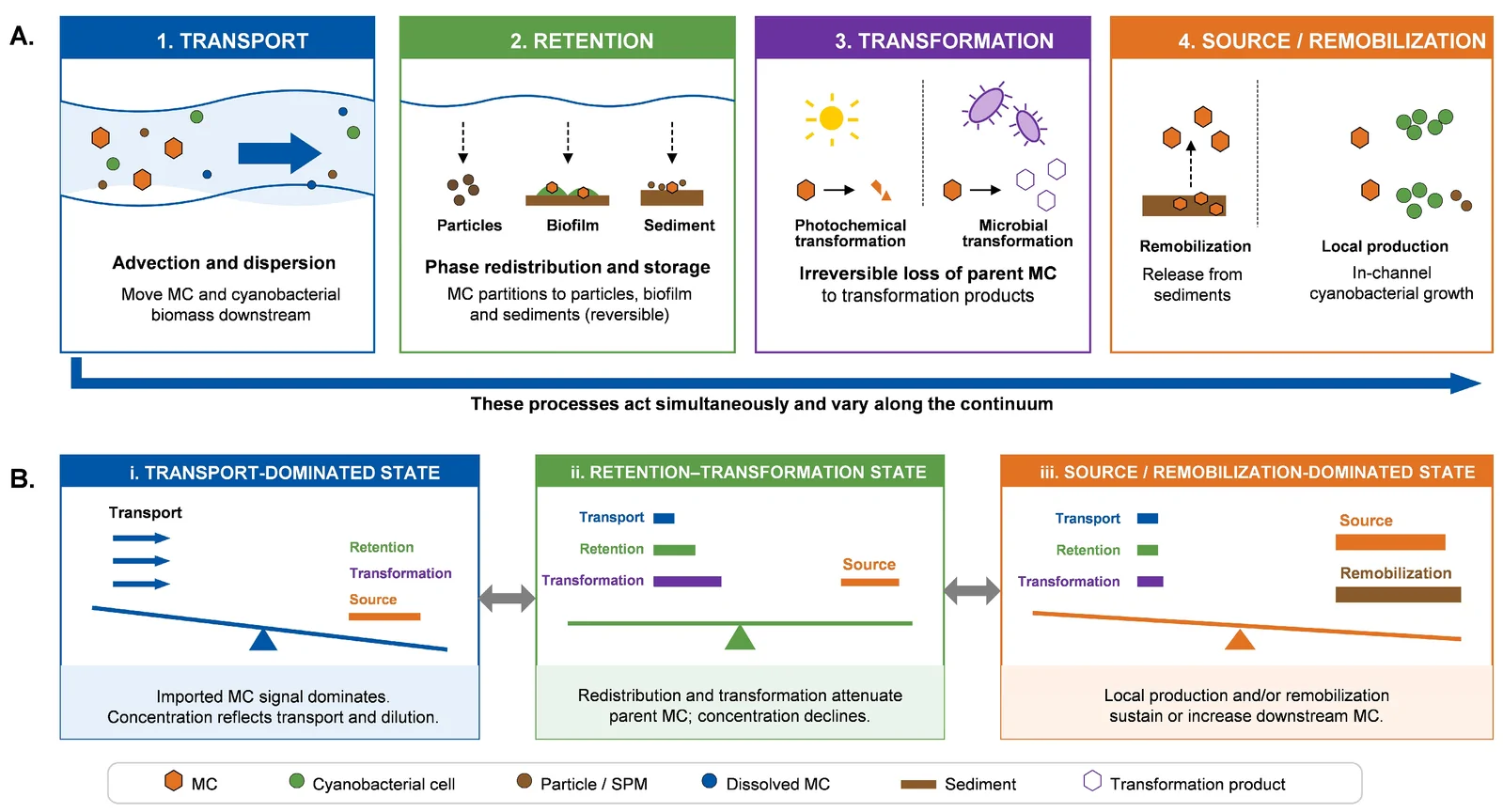
The lake–river continuum as a dynamic biogeochemical reactor for microcystins. (**A**) Four interacting processes regulate downstream MC fate: transport redistributes MC and cyanobacterial biomass; retention transfers MC among particles, biofilms, and sediments; photochemical and microbial transformation reduce parent MC; and local production or remobilization can renew the mobile MC signal. (**B**) Their relative dominance produces three conceptual reactor states: transport-dominated, retention–transformation, and source/remobilization-dominated. These states are dynamic rather than fixed river types and may shift with hydrological and environmental conditions. Arrows, bars, and balance symbols indicate relative process importance conceptually rather than quantitatively. Dashed arrows indicate transfer to retained phases in the retention panel and remobilization from sediment in the source/remobilization panel; dotted vertical lines separate paired subprocesses; and colors distinguish the four process classes: transport, retention, transformation, and source/remobilization.

Three conceptual operational states summarize this interaction; they are hypotheses about relative process dominance, not a validated classification of river types. In a transport-dominated state, short residence time limits reaction and much of the incoming MC mass remains mobile even if concentration is diluted. In a retention–transformation state, longer residence and stronger interfacial contact allow sorption, sediment exchange, photochemistry, and microbial degradation to alter phase distribution and parent-MC mass. In a source/remobilization-dominated state, local production or renewed release from cells, particles, or sediments maintains the mobile signal despite concurrent attenuation. These are dynamic states rather than fixed river types; the same reach can switch among them as discharge, bloom stage, temperature, turbidity, and management operations change. Together, these interacting processes and shifts in relative process dominance define the lake–river continuum as a dynamic biogeochemical reactor (Figure 2).

## 4. Changing Exposure Pathways and Ecological Effects

The ecological consequence of reactor processing depends on how phase redistribution changes uptake. Environmental availability, internal dose, and biological effect are distinct layers. Dissolved, cell-associated, particle-associated, sediment-associated, and biotic pools are not equivalent exposure matrices, and a decrease in dissolved MC can coincide with greater exposure of particle feeders, benthic organisms, or consumers of contaminated prey [20,35,39]. Tissue burdens can also integrate exposure over longer periods than a water sample, as demonstrated in estuarine bivalves downstream of freshwater blooms [10]. The relevant question is therefore not simply whether MC is present, but which matrix is accessible to a particular receptor and for how long.

### 4.1. Environmental Matrix, Uptake Route, and Internal Dose

Exposure needs to be matched to receptor. Dissolved MC is most relevant to direct water contact and uptake; cell- and particle-associated MC matter for grazers and filter feeders; sediments define a potential benthic pathway; and tissues reflect uptake, metabolism, and elimination. Detection in a matrix does not establish the internal dose delivered to an organism. Two organisms occupying the same reach can therefore receive different doses because they contact different phases, ingest different particles or prey, and differ in uptake and depuration. Conversely, the same organism may retain toxin after the external water signal has declined.

The toxicological mechanism reinforces this distinction. Inhibition of serine/threonine protein phosphatases, particularly PP1 and PP2A, is a well-characterized MC mechanism, with direct experimental evidence for MC-LR [18,49,59]. Oxidative stress and downstream physiological disruption have been reported across exposed taxa [60,61,62,63,64], but potency, uptake, and toxicokinetics should not be assumed to be identical among congeners. These responses provide useful mechanistic endpoints, but they are not specific indicators of exposure route. Biomarkers should therefore be interpreted together with chemical measurements and toxin phase rather than used as stand-alone evidence that a particular environmental compartment caused the observed effect.

### 4.2. Receptor-Specific Ecological Effects

The evidence below distinguishes purified MC-LR experiments from broader MC measurements and exposure to toxigenic cyanobacteria; congener-specific findings are not generalized to all MCs. Submerged macrophytes provide a useful lower-trophic example because they occupy the water–sediment interface. MC-LR can alter redox status, cellular structure, ion homeostasis, photosynthesis, and phytohormone-related pathways in *Vallisneria natans* [61,62,65], with sublethal responses to MC-LR also reported in *Ceratophyllum demersum* [66]. These endpoints do not, by themselves, demonstrate population-level impairment in a natural continuum. Chemical context can further modify response: combined MC-LR and phenanthrene exposure produced concentration-dependent antagonistic and synergistic effects in V. natans [67]. Other mixture studies likewise show that co-exposure effects vary with stressor, concentration, and endpoint [68,69,70], arguing against a universal assumption of synergy.

Zooplankton illustrate the importance of test material and population history. Exposure to toxigenic *Microcystis* can reduce *Daphnia* survival or reproduction [71], but whole-cyanobacterial exposure includes food-quality effects, morphology, and other metabolites in addition to MC. Dietary MC-LR experiments provide more toxin-specific evidence [72]. Natural populations can also adapt rapidly to toxic cyanobacteria [73], and parental cyanotoxin exposure can influence offspring after transfer to clean medium [74]. Consequently, continuum-scale population risk should not be inferred from a single clone, a single exposure route, or a short acute test.

Bivalves and fish illustrate tissue-integrated and dietary exposure. *Corbicula fluminea* accumulates MC during exposure to *Microcystis* [53], and bivalve tissue contamination can persist after the water-column signal weakens [10]. In fish, MC burdens are often higher in liver than muscle, although tissue distribution depends on species, route, and time [54,55]. Liver burden is relevant to organismal toxicity, whereas human dietary assessment depends more directly on edible tissues; the two questions should not be conflated. Bioaccumulation also does not imply biomagnification. Meta-analysis shows mixed trophic patterns [56], and an experimental food chain reported accumulation without consistent biomagnification [57].

### 4.3. Spatial Displacement and Temporal Integration

Continuum exposure can therefore be displaced in both space and time. MC can move longitudinally downstream, laterally into sediments and riparian food webs, and vertically through consumer pathways. Tissue retention in bivalves and fish and parental effects in zooplankton can extend biological exposure beyond the external pulse [10,54,55,74]. Field measurements also demonstrate transfer from aquatic insects to riparian consumers [58]. These observations expand the set of potential receptors, but they do not imply universal risk amplification: redistribution can increase exposure for one receptor while decreasing it for another, and exposure transfer is not itself proof of adverse effect. The most defensible continuum interpretation links environmental matrix to uptake route, internal dose, receptor, and endpoint.

## 5. Continuum-Aware Risk Assessment and Management

The continuum does not require a new ecological risk-assessment paradigm; it requires conventional assessment to represent exposure more faithfully. The recurring limitation is the use of a single water-column concentration as a universal surrogate despite changes in hydrology, toxin phase, uptake route, and receptor. A continuum-aware assessment therefore retains standard problem formulation, exposure assessment, effects assessment, and risk characterization while resolving where, when, and through which matrix exposure occurs.

### 5.1. Limitations of Concentration-Based Assessment

Human-health and ecological benchmarks should not be conflated. The 2026 WHO Guidelines retain provisional drinking-water values of 1 µg/L for lifetime exposure and 12 µg/L for short-term exposure to total microcystins, defined as the sum of all congeners in free and cell-bound forms [75]. Here, cell-bound means associated with cyanobacterial cells, not covalently bound to tissue proteins. The short-term value is derived from an MC-LR mouse-study no-observed-adverse-effect level of 40 µg/kg body weight per day, an uncertainty factor of 100, a 60 kg adult, and 2 L/day drinking-water consumption with 100% exposure allocation to water. It applies for up to about two weeks while corrective treatment is implemented, not repeated seasonal exceedance. WHO also advises alternative safe water for bottle-fed infants and small children above 3 µg/L, even briefly [75]. These are human-health values, not ecological water-quality criteria.

Conversely, sublethal responses below 1 µg/L do not, by themselves, demonstrate population-level impairment. Species-sensitivity distributions can support ecological screening [76], but existing MC-LR applications rely heavily on acute toxicity data; chronic, receptor-specific effect thresholds remain a more important gap than simply deriving a lower analogue of the drinking-water value.

Water concentration alone is also an incomplete exposure metric when MC is redistributed among cells, particles, sediments, and tissues [20,35,53,55]. The direction of bias is receptor-dependent: sorption can reduce dissolved exposure while increasing ingestion-based exposure, sediment storage can create a potential benthic pathway without proving delivery of a bioavailable dose, and tissue retention can outlast the water signal. Composition adds further uncertainty because congener-specific transformation can change mixture structure during transport [22]. Co-occurring contaminants can produce antagonistic, additive, or synergistic effects in laboratory studies [67,68,69,70], while existing cyanotoxin mixture models primarily predict co-occurrence and human-exposure screening rather than ecological joint effects [77].

Hydrological state makes exposure non-stationary. High discharge may combine lower concentration with greater exported mass, whereas low flow can increase residence time, transformation opportunity, and local bloom development. Climate and nutrient conditions modify the frequency of these states [26,27,78], but they are not direct proxies for downstream risk. The relevant exposure metric depends on the endpoint: acute effects may track short peaks, chronic effects concentration–duration history, and dietary exposure accumulation and depuration. Fixed-interval monitoring can therefore miss risk windows created by bloom senescence, storms, drought, or managed releases [28,29,33].

### 5.2. A Reach–Pathway–Receptor Framework

A practical assessment unit is reach × pathway × receptor. For each unit, the minimum information is the dominant source or hydraulic state, the relevant MC matrix, the plausible uptake route, exposure duration, the receptor and effect benchmark, and major uncertainty. This structure makes explicit where water concentration is an adequate surrogate and where it is not. It also prevents a high concentration in one compartment from being interpreted automatically as high risk to every receptor.

A tiered approach can increase resolution only when needed. For Tier 1, we propose a core set of paired whole-water total MC and dissolved MC measurements, cyanobacterial cell abundance or biovolume with community composition, and discharge. Total-water analysis requires validated cell disruption and extraction, while the dissolved fraction is measured after gentle separation that minimizes cell lysis [5,75]. The difference between total and dissolved concentrations is only an operational estimate of cell- plus particle-associated MC; it should not automatically be labelled intracellular toxin. Where phase partitioning is important, retained cells and particles should be extracted separately, with filter size, recovery, congener coverage, and detection limits reported [5,35].

Sampling should connect the source lake, outlet or export boundary, downstream mixing reaches, retention zones, and receptor-relevant sites. Concentration and discharge should be paired at the same cross-section and time; longitudinal comparisons should account for travel time and tributary inputs. Routine sampling should be supplemented during bloom decline, storms, prolonged low flow, and managed releases, because a fixed schedule may miss export and exposure pulses [28,29,33]. This design distinguishes concentration changes from mass export without assuming that all downstream decreases represent removal.

Tier 2 adds measurements according to the suspected process or exposure route. Suspended-particle concentrations and particle-associated MC are useful in turbid transport reaches; surface sediment and pore-water MC are appropriate where deposition or remobilization is plausible [35,39]. Bivalves or other relevant biota can integrate exposure when dietary or persistent tissue burdens are the concern [10,34,53,54,55]. Tissue measurements should specify the organ, wet- or dry-mass basis, and whether the method targets extractable parent congeners, conjugates, or an integrated free-plus-bound pool [50,51,52]. No single matrix needs to be measured at every site.

Tier 3 links these chemical observations to chronic or receptor-specific effect data, uptake and depuration, and relevant mixtures. Product screening and endpoint-specific bioassays are particularly useful where treatment or in-stream transformation is being interpreted as detoxification [42,49]. The desired output is not one continuum-wide risk quotient, but a spatial and temporal account of where exposure changes, which pathway and receptor are involved, and how strongly an adverse-effect interpretation is supported.

### 5.3. Research Priorities

Three priorities would most directly test the continuum framework. First, close field mass balances using the paired, phase-resolved design outlined in Section 5.2, with sufficient temporal coverage to resolve export events rather than only source-lake bloom maxima [13,28,29]. Additional matrices should answer a defined mass-balance or exposure question rather than be measured everywhere by default. Molecular indicators such as mcy genes can strengthen early warning of toxigenic populations, but their relationship with toxin concentration is system-dependent and they should complement rather than replace chemical measurements [79,80].

Second, quantify process rates under field conditions. Short-reach mass balances, conservative tracers, residence-time estimates, and measurements of light, DOM, SPM, sediment exchange, and microbial activity can separate reversible retention from irreversible transformation and from production or remobilization. Such measurements would turn the three reactor states into testable hypotheses [19,39]. Third, link molecular and predictive tools to these measurable rates. Omics studies reveal diverse microbial responses during MC degradation [45,46,81], but genes or transcripts alone do not establish in situ flux. Predictive models should therefore begin with source flux, discharge, travel time, and major gain/loss terms, adding process modules only when observations justify them.

Existing monitoring and early-warning frameworks demonstrate the value of high-frequency observations and hydrodynamic simulation [82]. At the continuum scale, model evaluation should focus on the timing and magnitude of downstream MC concentration and mass-flux peaks rather than only bloom extent in the source lake. The ecological evidence base needs the same selectivity: chronic tests should emphasize receptors and exposure routes that are plausible in connected river networks, and tissue burdens should be related to effects rather than treated as risk endpoints by themselves.

### 5.4. Management Implications

Management remains hierarchical. Nutrient reduction and bloom control at the source are the primary long-term means of reducing MC available for export, while downstream surveillance should focus on reaches where transport, storage, or local production changes the source-to-receptor pathway. Existing bloom-monitoring and forecasting programmes provide a strong foundation [7,82], but trigger-based surveillance becomes more informative when linked to source activity and hydraulic state rather than a fixed seasonal schedule.

Hydraulic infrastructure should be treated as a controllable boundary condition, not an assumed mitigation tool. Pumping, sluice operation, reservoir release, or channel modification may reduce local concentration yet increase exported mass or relocate exposure. Before–after measurements or scenario-based mass balances are therefore needed before operational changes are interpreted as toxin-control measures. Biofilm-rich engineered systems and constructed wetlands are promising for cyanotoxin biodegradation [48], but field-scale performance, hydraulic loading, transformation products, and storage or remobilization remain unresolved. The practical goal is to identify a limited set of risk transitions where intervention reduces exposure rather than merely moving it downstream.

## 6. Conclusions

Viewing microcystins through the lake–river continuum shifts the unit of analysis from a bloom-affected lake to a connected source-to-receptor system. River reaches are neither passive conduits nor intrinsically effective attenuation zones. Their role depends on the balance among transport, reversible retention, irreversible transformation, local production, and remobilization. The resulting downstream signal can therefore change in concentration, phase distribution, parent-toxin mass, and biological availability without those changes occurring in parallel.

The framework also separates three quantities that are often conflated: the source pool, export flux, and receptor exposure. Hydrology determines reaction opportunity; particles, sediments, biofilms, and biota redistribute exposure; photochemical and microbial reactions can reduce parent-MC mass; and stored or newly produced toxin can renew the mobile signal. Consequently, a decline in dissolved MC is not sufficient evidence of detoxification, and water-column concentration is not a universal exposure metric.

The strongest next steps are field-resolved mass balances, direct measurements of process rates, and chronic effect data matched to realistic exposure routes. Source control remains primary, while downstream monitoring and hydraulic management should be targeted to reaches where the source-to-receptor pathway demonstrably changes. The central question is no longer simply how far MC travels from a bloom but where, when, and through which processes transport changes ecological exposure and risk.

## Figures and Tables

**Table 1:** Representative hydrological, cyanobacterial, and microcystin evidence across connected waters. Regulated-river comparisons provide supporting process evidence rather than a measured source-lake export transect.

System/Study	Hydrological Context	Cyanobacterial Evidence	MC Metric and Matrix	Evidence/Inference	Main Limitation
Taihu–connected rivers Wan et al. [13]	Connected lentic and lotic compartments; contrasting flow conditions.	Lake *Microcystis* >90% relative abundance; river assemblages included *Oscillatoria* and *Dolichospermum*.	Intracellular MC: lake maximum 14.5 µg/L; river values generally <1.0 µg/L.	Field survey: community and toxin heterogeneity across compartments.	Spatial association, not a phase-resolved export balance or an isolated flow–richness effect.
Milford Lake–Republican River–Kansas River 2011; Graham et al. [28]	Reservoir-release event; observations over 173 miles (approximately 278 km).	Cyanobacterial abundance measured; abundance was not a reliable toxin surrogate.	Total MC: outlet ≤20 µg/L; Republican River >1 µg/L during most of September.	Event observations distinguish source-lake, outlet, and downstream conditions.	Deviation from simple dilution predictions was not statistically tested; separate loss processes unresolved.
Copco/Iron Gate–Klamath River–estuary 2012; Otten et al. [32]	Historical reservoir–river configuration; downstream tracking over approximately 300 km.	*Microcystis*; molecular markers used for source tracking.	Total MC: 165 µg/L in Copco Reservoir; 3.6 µg/L in the lower estuary.	Molecular and chemical observations support distant source-to-receiver connectivity.	Site concentrations do not describe a matched water parcel; no removal percentage follows from the endpoints.
Thames–St. Clair–Detroit–Erie 2016–2019; Crevecoeur et al. [31]	Detroit River mean discharge 6383 m^3^/s; hydrological background, not a toxin-paired measurement.	Cyanobacterial sequence variants document dispersal and environmental selection.	Water-column microbial DNA; no analytical MC concentration outcome.	Sequencing supports biological connectivity across connected waters.	Shared taxa or sequence variants are not measurements of MC mass flux or standardized species richness.
Regulated Moselle 2017–2020; Kleinteich et al. [29]	June–September discharge: 69–76% of corresponding 1997–2016 reference medians.	Recurrent *Microcystis aeruginosa* blooms.	Intracellular MC, filter extracts: median 0.17 µg/L in August; 0.11 µg/L in September.	Field comparison associates blooms with warm, low-discharge summers.	Dissolved MC and imported versus local toxin were not resolved; no universal flow threshold.
Regulated Moselle 2021 versus 2022; Klotz et al. [33]	Matched summer means: 328 versus 39 m^3^/s; modelled flow time 9.9 versus 31.8 days.	*Microcystis* bloom in the drier, warmer 2022.	No analytical MC concentrations reported; biomass is not toxin concentration.	Field monitoring and modelling contrast wet and drought conditions.	Temperature and nutrients covaried; flow data cannot be paired with MC values from the separate 2017–2020 study.
Freshwater lakes–outlets–Puget Sound Preece et al. [34]	Lake outflows enter coastal receiving waters.	Bloom-affected source lakes; no standardized flow–richness result inferred.	ELISA water maxima: source lakes 2700 µg/L; marine water 0.34 µg/L. MC also detected in mussels.	Chemical measurements and LC–MS/MS confirmation support cross-boundary exposure and tissue uptake.	Water maxima are not paired dilution endpoints; tissue detection alone does not establish an adverse effect.

Note: Values retain their original fractions and summary statistics (maxima, medians, or period means); they are not harmonized effect sizes. Discharge (m^3^/s), velocity (m/s), and modelled flow time (days) are different quantities. Qualitative hydrological descriptions do not imply that the original study lacked flow measurements. Community composition and abundance are not species richness. The table is a descriptive synthesis, not a paired concentration–discharge dataset. MC, microcystin.

**Table 2:** Major continuum processes, their implications for microcystin fate and exposure, and the principal uncertainty for field application.

Process/Function	Representative Evidence	Change in MC Fate	Exposure Implication	Main Uncertainty	Refs.
Hydrological transport	Taihu–Liangxi biomass modelling; Kansas release observations.	Redistribution; mass export depends jointly on discharge and concentration.	Source-lake and downstream concentrations need not coincide.	Phase-resolved fluxes and process-specific losses during hydraulic operations.	[9,13,28,30]
Connected-water dispersal	Great Lakes sequencing; Klamath chemical and molecular source tracking.	Cells and toxin-production potential move among connected habitats.	Distant source populations can contribute to downstream exposure.	Biological connectivity alone does not quantify toxin flux.	[31,32]
Reversible retention	SPM–biofilm experiments and particle sorption studies.	MC shifts between dissolved and retained pools.	Lower dissolved exposure can coincide with greater particle ingestion.	Partitioning and retention fluxes under natural river conditions.	[20,39,40]
Sediment storage/remobilization	Sediment and pore-water measurements; benthic *Microcystis* recruitment.	Storage can delay return to mobile pools.	Potential benthic exposure or renewed water-column signal.	Storage does not establish net sediment-to-water flux or bioavailable dose.	[20,35,36,37,38]
Photochemical transformation	Natural-light and surface-water experiments.	Parent MC undergoes direct or indirect phototransformation.	Parent-MC decline does not establish complete detoxification.	Whole-reach rates, product identity, and residual bioactivity under variable optics and DOM.	[41,42,43,44]
Microbial degradation	Lake communities, beach sand, biofilms, and wetland mesocosms.	Biotransformation can couple with interfacial retention.	Reactive interfaces can reduce mobile parent MC.	In situ rates, non-mlr pathways, and the fate and activity of products.	[14,45,46,47,48]
In-channel production	Warm, low-discharge Moselle bloom observations.	Local growth can add MC to transported inventories.	Downstream concentration may include local and imported toxin.	The two contributions and their prevalence across rivers remain unresolved.	[29,33]
Biological uptake	Bivalves, fish, and aquatic-to-riparian food webs.	MC is transferred to tissues and integrated over time.	Tissue burdens can persist after water concentrations decline.	Links between tissue burden, bioavailable dose, and adverse effects; no automatic biomagnification.	[10,34,53,54,55,56,57,58]

Note: Evidence types identify what the cited studies demonstrate; they are not quality scores. Process-specific observations do not establish their relative contribution in every continuum. MC, microcystin; SPM, suspended particulate matter; DOM, dissolved organic matter.

## Data Availability

No new data were created or analyzed in this review. Data sharing is not applicable to this article.

## References

[1] Wang Y., Zhao D., Woolway R.I., Yan H., Paerl H.W., Zheng Y., Zheng C., Feng L. (2025). Global elevation of algal bloom frequency in large lakes over the past two decades. Natl. Sci. Rev..

[2] Ho J.C., Michalak A.M., Pahlevan N. (2019). Widespread global increase in intense lake phytoplankton blooms since the 1980s. Nature.

[3] Feng L., Wang Y., Hou X., Qin B., Kutser T., Qu F., Chen N., Paerl H.W., Zheng C. (2024). Harmful algal blooms in inland waters. Nat. Rev. Earth Environ..

[4] Huisman J., Codd G.A., Paerl H.W., Ibelings B.W., Verspagen J.M.H., Visser P.M. (2018). Cyanobacterial blooms. Nat. Rev. Microbiol..

[5] Chorus I., Welker M. (2021). Toxic Cyanobacteria in Water: A Guide to Their Public Health Consequences, Monitoring and Management.

[6] Villalobos T., Suárez-Isla B., Garcia C. (2025). Health and Environmental Impacts of Cyanobacteria and Cyanotoxins from Freshwater to Seawater. Toxins.

[7] Qin B., Li W., Zhu G., Zhang Y., Wu T., Gao G. (2015). Cyanobacterial bloom management through integrated monitoring and forecasting in large shallow eutrophic Lake Taihu (China). J. Hazard. Mater..

[8] Guo L. (2007). Doing battle with the green monster of Taihu Lake. Science.

[9] He X., Wang H., Zhuang W., Liang D., Ao Y. (2021). Risk prediction of microcystins based on water quality surrogates: A case study in a eutrophicated urban river network. Environ. Pollut..

[10] Wallace M.K., Kudela R.M., Gobler C.J. (2025). Microcystin contamination of shellfish along the freshwater-to-marine continuum within US mid-Atlantic and Northeast estuaries. Harmful Algae.

[11] Miller M.A., Kudela R.M., Mekebri A., Crane D., Oates S.C., Tinker M.T., Staedler M., Miller W.A., Toy-Choutka S., Dominik C. (2010). Evidence for a novel marine harmful algal bloom: Cyanotoxin (microcystin) transfer from land to sea otters. PLoS ONE.

[12] Fraga M., Churro C., Leão-Martins J., Rudnitskaya A., Botelho M.J. (2025). Cyanotoxins on the move—Freshwater origins with marine consequences: A systematic review of global changes and emerging trends. Mar. Pollut. Bull..

[13] Wan X., Li Y., Xue Q., Wang G., Xie L. (2026). Functional divergence and toxin coupling of cyanobacterial blooms across the lake–river continuum: Insights from the Lake Taihu watershed. Toxins.

[14] Dziga D., Wasylewski M., Wladyka B., Nybom S., Meriluoto J. (2013). Microbial degradation of microcystins. Chem. Res. Toxicol..

[15] Tan Y., Wang Y., Bing X., Jiang J., Guo G., Cui F., Wang K., Meng Z., Liu Y., Zhu Y. (2025). Coupling effect of cyanobacterial blooms with migration and transformation of typical pollutants in lake or reservoir: Enhanced or decreased?. Environ. Res..

[16] Spoof L., Catherine A., Meriluoto J., Spoof L., Codd G.A. (2017). Appendix 3: Tables of microcystins and nodularins. Handbook of Cyanobacterial Monitoring and Cyanotoxin Analysis.

[17] Meriluoto J., Blaha L., Bojadzija G., Bormans M., Brient L., Codd G.A., Drobac D., Faassen E.J., Fastner J., Hiskia A. (2017). Toxic cyanobacteria and cyanotoxins in European waters—Recent progress achieved through the CYANOCOST Action and challenges for further research. Adv. Oceanogr. Limnol..

[18] Buratti F.M., Manganelli M., Vichi S., Stefanelli M., Scardala S., Testai E., Funari E. (2017). Cyanotoxins: Producing organisms, occurrence, toxicity, mechanism of action and human health toxicological risk evaluation. Arch. Toxicol..

[19] Lerminiaux J., Bogard M.J., Leavitt P.R., Vogt R.J., Finlay K. (2026). Multiscale regulation of microcystin production, persistence and degradation in inland waters. Nat. Water.

[20] Shingai Q.K., Wilkinson G.M. (2023). Microcystin as a biogeochemical cycle: Pools, fluxes, and fates of the cyanotoxin in inland waters. Limnol. Oceanogr. Lett..

[21] de Maagd P.G.J., Hendriks A.J., Seinen W., Sijm D.T.H.M. (1999). pH-dependent hydrophobicity of the cyanobacterial toxin microcystin-LR. Water Res..

[22] Hu C., Zuo Y., Peng L., Gan N., Song L. (2021). Widespread Distribution and Adaptive Degradation of Microcystin Degrader (mlr-Genotype) in Lake Taihu, China. Toxins.

[23] Paerl H.W., Gardner W.S., Havens K.E., Joyner A.R., McCarthy M.J., Newell S.E., Qin B., Scott J.T. (2016). Mitigating cyanobacterial harmful algal blooms in aquatic ecosystems impacted by climate change and anthropogenic nutrients. Harmful Algae.

[24] Chaffin J.D., Westrick J.A., Furr E., Birbeck J.A., Reitz L.A., Stanislawczyk K., Li W., Weber P.K., Bridgeman T.B., Davis T.W. (2022). Quantification of microcystin production and biodegradation rates in the western basin of Lake Erie. Limnol. Oceanogr..

[25] Saleem F., Jiang J.L., Li E., Tran K., Boere A., Rahman M., Paschos A., Westrick J.A., Zastepa A., Edge T.A. (2024). Regional and longitudinal dynamics of cyanobacterial blooms/cyanobiome and cyanotoxin production in the Great Lakes area. Toxins.

[26] Wang C., Wang M., Xie M., Qi L., Chen M., Song X., Yin J., Wei Y., Xu M., Pan L. (2026). Climate extremes intensify global lake eutrophication by increasing the stress resistance of harmful bloom-forming algae. Nat. Commun..

[27] Merder J., Harris T., Zhao G., Stasinopoulos D.M., Rigby R.A., Michalak A.M. (2023). Geographic redistribution of microcystin hotspots in response to climate warming. Nat. Water.

[28] Graham J.L., Ziegler A.C., Loving B.L., Loftin K.A. (2012). Fate and Transport of Cyanobacteria and Associated Toxins and Taste-and-Odor Compounds from Upstream Reservoir Releases in the Kansas River, Kansas, September and October 2011.

[29] Kleinteich J., Frassl M.A., Schulz M., Fischer H. (2024). Climate change triggered planktonic cyanobacterial blooms in a regulated temperate river. Sci. Rep..

[30] He X., Wang H., Fan L., Liang D., Ao Y., Zhuang W. (2020). Quantifying physical transport and local proliferation of phytoplankton downstream of an eutrophicated lake. J. Hydrol..

[31] Crevecoeur S., Edge T.A., Watson L.C., Watson S.B., Greer C.W., Ciborowski J.J.H., Diep N., Dove A., Drouillard K.G., Frenken T. (2023). Spatio-temporal connectivity of the aquatic microbiome associated with cyanobacterial blooms along a Great Lake riverine-lacustrine continuum. Front. Microbiol..

[32] Otten T.G., Crosswell J.R., Mackey S., Dreher T.W. (2015). Application of molecular tools for microbial source tracking and public health risk assessment of a *Microcystis* bloom traversing 300 km of the Klamath River. Harmful Algae.

[33] Klotz F., Herrmann M., Ishikawa M., Helms M., Brehm T., Baschek B., Fischer H., Kleinteich J. (2026). Spatio-temporal dynamics of riverine cyanobacteria and selected water quality indicators under two hydrological regimes. Sci. Rep..

[34] Preece E.P., Moore B.C., Hardy F.J. (2015). Transfer of microcystin from freshwater lakes to Puget Sound, WA and toxin accumulation in marine mussels (*Mytilus trossulus*). Ecotoxicol. Environ. Saf..

[35] Zastepa A., Pick F.R., Blais J.M., Saleem A. (2015). Analysis of intracellular and extracellular microcystin variants in sediments and pore waters. Anal. Chim. Acta.

[36] Cheng C., Steinman A.D., Zhang K., Lin Q., Xue Q., Wang X., Xie L. (2023). Risk assessment and identification of factors influencing the historical concentrations of microcystin in Lake Taihu, China. J. Environ. Sci..

[37] Verspagen J.M.H., Snelder E.O.F.M., Visser P.M., Huisman J., Mur L.R., Ibelings B.W. (2004). Recruitment of benthic *Microcystis* (Cyanophyceae) to the water column: Internal buoyancy changes or resuspension?. J. Phycol..

[38] Romanis C.S., Timms V.J., Nebauer D.J., Crosbie N.D., Neilan B.A. (2024). Microbiome analysis reveals *Microcystis* blooms endogenously seeded from benthos within wastewater maturation ponds. Appl. Environ. Microbiol..

[39] Tang B., Hung W., Salam M., Zhang L., Yang Y., Niu J., Li H., Zhang L. (2024). *Suspended particulate* matter-biofilm aggregates benefit microcystin removal in turbulent water but trigger toxicity toward *Daphnia magna*. Water Res..

[40] Shi Y., Almuhtaram H., Andrews R.C. (2023). Adsorption of per- and polyfluoroalkyl substances (PFAS) and microcystins by virgin and weathered microplastics in freshwater matrices. Polymers.

[41] Wörmer L., Huerta-Fontela M., Cirés S., Carrasco D., Quesada A. (2010). Natural photodegradation of the cyanobacterial toxins microcystin and cylindrospermopsin. Environ. Sci. Technol..

[42] Feng J., Kiki C., Li X., Sun Q., Zhao F. (2025). Deciphering the natural attenuation of cyanotoxins: Dissipation, transformation pathways, and genotoxicity. Environ. Health.

[43] Kurtz T., Zeng T., Rosario-Ortiz F.L. (2021). Photodegradation of cyanotoxins in surface waters. Water Res..

[44] Wenk J., Graf C., Aeschbacher M., Sander M., Canonica S. (2021). Effect of solution pH on the dual role of dissolved organic matter in sensitized pollutant photooxidation. Environ. Sci. Technol..

[45] Krausfeldt L.E., Steffen M.M., McKay R.M., Bullerjahn G.S., Boyer G.L., Wilhelm S.W. (2019). Insight Into the Molecular Mechanisms for Microcystin Biodegradation in Lake Erie and Lake Taihu. Front. Microbiol..

[46] Salter C., Westrick J.A., Chaganti S.R., Birbeck J.A., Peraino N.J., Weisener C.G. (2023). Elucidating microbial mechanisms of microcystin-LR degradation in Lake Erie beach sand through metabolomics and metatranscriptomics. Water Res..

[47] Babica P., Bláha L., Maršálek B. (2005). Removal of microcystins by phototrophic biofilms. A microcosm study. Environ. Sci. Pollut. Res..

[48] Thyssen L.A., Martinez i Quer A., Arias C.A., Ellegaard-Jensen L., Carvalho P.N., Johansen A. (2024). Constructed wetland mesocosms improve the biodegradation of microcystin-LR and cylindrospermopsin by indigenous bacterial consortia. Harmful Algae.

[49] MacKintosh C., Beattie K.A., Klumpp S., Cohen P., Codd G.A. (1990). Cyanobacterial microcystin-LR is a potent and specific inhibitor of protein phosphatases 1 and 2A from both mammals and higher plants. FEBS Lett..

[50] Kohoutek J., Procházková T., Adamovský O., Palíková M., Hilscherová K. (2019). Stable-isotope dilution LC-MS/MS method for quantitative determination of microcystin conjugates with cysteine and glutathione in biotic matrices. Anal. Bioanal. Chem..

[51] Miles C.O., Sandvik M., Nonga H.E., Ballot A., Wilkins A.L., Rise F., Jaabaek J.A.H., Loader J.I. (2016). Conjugation of microcystins with thiols is reversible: Base-catalyzed deconjugation for chemical analysis. Chem. Res. Toxicol..

[52] Bouteiller P., Biré R., Foss A.J., Guérin T., Lance E. (2024). Analysis of total microcystins by Lemieux oxidation and liquid chromatography-mass spectrometry in fish and mussels tissues: Optimization and comparison of protocols. Sci. Total Environ..

[53] Zhang J., Yu M., Gao Y., Zhang M., Dong J., Li M., Li X. (2023). Feeding behavior, microcystin accumulation, biochemical response, and ultramicrostructure changes in edible freshwater bivalve *Corbicula fluminea* exposed to *Microcystis aeruginosa*. Environ. Sci. Pollut. Res..

[54] Adamovský O., Kopp R., Hilscheróvá K., Babica P., Palíková M., Pašková V., Navrátil S., Marsálek B., Bláha L. (2007). Microcystin kinetics (bioaccumulation and elimination) and biochemical responses in common carp (*Cyprinus carpio*) and silver carp (*Hypophthalmichthys molitrix*) exposed to toxic cyanobacterial blooms. Environ. Toxicol. Chem..

[55] Peng L., Tang Q., Gu J., Lei L., Chen W., Song L. (2020). Seasonal variation of microcystins and their accumulation in fish in two large shallow lakes of China. Ecotoxicology.

[56] Kozlowsky-Suzuki B., Wilson A.E., Ferrão-Filho A.d.S. (2012). Biomagnification or biodilution of microcystins in aquatic foodwebs? Meta-analyses of laboratory and field studies. Harmful Algae.

[57] Glidewell D.R., Beyer J.E., Hambright K.D. (2025). *Microcystins bioaccumulate* but do not biomagnify in an experimental aquatic food chain. Harmful Algae.

[58] Moy N.J., Dodson J., Tassone S.J., Bukaveckas P.A., Bulluck L.P. (2016). Biotransport of algal toxins to riparian food webs. Environ. Sci. Technol..

[59] Rajpoot R., Rajput S., Koiri R.K. (2025). Microcystin-LR and its health impacts: Chemistry, transmission routes, mechanisms of toxicity and target organs. Toxicol. Rep..

[60] Lin W., Hung T.-C., Kurobe T., Wang Y., Yang P. (2021). Microcystin-induced immunotoxicity in fishes: A scoping review. Toxins.

[61] Jiang J., Gu X., Song R., Wang X., Yang L. (2011). Microcystin-LR induced oxidative stress and ultrastructural alterations in mesophyll cells of submerged macrophyte *Vallisneria natans* (Lour.) Hara. J. Hazard. Mater..

[62] Cheng C., Steinman A.D., Xue Q., Wan X., Xie L. (2023). The disruption of calcium and hydrogen ion homeostasis of submerged macrophyte *Vallisneria natans* (Lour.) Hara caused by microcystin-LR. Aquat. Toxicol..

[63] Chen L., Wang Y., Giesy J.P., Chen F., Shi T., Chen J., Xie P. (2018). Microcystin-LR affects the hypothalamic-pituitary-inter-renal (HPI) axis in early life stages (embryos and larvae) of zebrafish. Environ. Pollut..

[64] Li Y., Yang H., Fu B., Kaneko G., Li H., Tian J., Wang G., Wei M., Xie J., Yu E. (2024). Integration of Multi-Omics, Histological, and Biochemical Analysis Reveals the Toxic Responses of Nile Tilapia Liver to Chronic Microcystin-LR Exposure. Toxins.

[65] Li Q., Tian X., Gu P., Yang G., Deng H., Zhang J., Zheng Z. (2022). Transcriptomic analysis reveals phytohormone and photosynthetic molecular mechanisms of a submerged macrophyte in response to microcystin-LR stress. Aquat. Toxicol..

[66] Dong J., Dai D., Yang Y., Wang F., Li X., Yuan J., Chen Y., Gao Y., Zhang M., Gao X. (2022). Responses of submerged macrophyte *Ceratophyllum demersum* to the gradient concentrations of microcystin-LR (MC-LR). Environ. Sci. Pollut. Res..

[67] Wan X., Zhang Y., Li Y., Yang F., Xie L. (2025). Phenanthrene amplifies microcystin-induced toxicity in the submerged macrophyte Vallisneria natans. Toxins.

[68] Lin W., Liao L., Ling L., Luo H., Jiang Y., Li X., Yao Y., Yang P. (2025). Combined effects of co-exposure to microcystin-LR and polystyrene microplastics on growth, brain pathology and thyroid hormone homeostasis in adult zebrafish. Ecotoxicol. Environ. Saf..

[69] Lin W., Luo H., Wu J., Liu X., Cao B., Liu Y., Yang P., Yang J. (2023). Polystyrene microplastics enhance the microcystin-LR-induced gonadal damage and reproductive endocrine disruption in zebrafish. Sci. Total Environ..

[70] Reddy S.G., Muskovac M.D., Alduis A., Manns J.C., Awali S., Hanna A., Jacob L.L., Ibrahim P., Alsharifi H., Vosbigian G. (2025). Combined exposure to ecologically relevant concentrations of atrazine and microcystin causes morphological changes in the hepatopancreas of crayfish. Integr. Comp. Biol..

[71] Martínez-Jerónimo F., Gonzalez-Trujillo L., Hernández-Zamora M. (2024). Continuous and intermittent exposure to the toxigenic cyanobacterium *Microcystis* aeruginosa differentially affects the survival and reproduction of *Daphnia curvirostris*. Toxins.

[72] Kim E.J., Jeon D., Park Y.J., Woo H., Eyun S.-I. (2024). Dietary exposure of the water flea *Daphnia galeata* to microcystin-LR. Anim. Cells Syst..

[73] Fajgenblat M., Gouwy E., Coone M., Almeida R., Boudry A., Cuypers K., van den Berg E., Vanoverberghe I., De Meester L., Decaestecker E. (2025). Rapid temporal adaptation structures tolerance to toxic cyanobacteria in a natural population of the water flea *Daphnia*. Evol. Lett..

[74] Dao T.S., Do-Hong L., Wiegand C. (2010). Chronic effects of cyanobacterial toxins on *Daphnia magna* and their offspring. Toxicon.

[75] World Health Organization (2026). Guidelines for Drinking-Water Quality: Fourth Edition Incorporating the First, Second and Third Addenda.

[76] Niu Z., Du L., Li J., Zhang Y., Lv Z. (2018). Ecological risk assessment of microcystin-LR in the upstream section of the Haihe River based on a species sensitivity distribution model. Chemosphere.

[77] Christensen V.G., Stelzer E.A., Eikenberry B.C., Olds H.T., LeDuc J.F., Maki R.P., Saley A.M., Norland J., Khan E. (2021). Cyanotoxin mixture models: Relating environmental variables and toxin co-occurrence to human exposure risk. J. Hazard. Mater..

[78] Lürling M., van Oosterhout F., Faassen E. (2017). Eutrophication and warming boost cyanobacterial biomass and microcystins. Toxins.

[79] Duan X., Zhang C., Struewing I., Li X., Allen J., Lu J. (2022). Cyanotoxin-encoding genes as powerful predictors of cyanotoxin production during harmful cyanobacterial blooms in an inland freshwater lake: Evaluating a novel early-warning system. Sci. Total Environ..

[80] Lu J., Struewing I., Wymer L., Tettenhorst D., Shoemaker J., Allen J. (2020). Use of qPCR and RT-qPCR for monitoring variations of microcystin producers and as an early warning system to predict toxin production in an Ohio inland lake. Water Res..

[81] Le V.V., Tran Q.G., Ko S.R., Oh H.M., Ahn C.Y. (2024). Insights into cyanobacterial blooms through the lens of omics. Sci. Total Environ..

[82] Qiu Y., Huang J., Luo J., Xiao Q., Shen M., Xiao P., Peng Z., Jiao Y., Duan H. (2025). Monitoring, simulation and early warning of cyanobacterial harmful algal blooms: An upgraded framework for eutrophic lakes. Environ. Res..

